# College and Hospital Notes

**Published:** 1900-06

**Authors:** 


### COLLEGE AND HOSPITAL NOTES.

THE 63d annual commencement exercises of the Medical College of Virginia, were held at the Academy of Music, Richmond, Thursday evening, May 10, 1900.

Dr. Christopher Tompkins, dean of the medical faculty, presided and conferred degrees upon 47 graduates. Of this number 40 received the degree of doctor of medicine, five, that of doctor of dental surgery, and two were graduates in pharmacy.

Dr. Charles A. L. Reed, of Cincinnati, O., delivered the address to the graduates. At the conclusion of the graduating exercises a banquet was given at the Jefferson Hotel, at which were assembled the graduating class, the board of visitors, the faculty, the alumni, and a number of invited guests.

The dinner was served in the style that has made the Jefferson famous. The toast-card was somewhat lengthy, but this was not true of any of the responses.

Dr. J. B. McCaw acted as toastmaster for a portion of the time, and then called Dr. George Ben Johnston to the chair.

The following were the toasts and speakers: Hon. Beverley T. Crump, "The Board of Visitors;" Dr. Robert F. Williams, "The Faculty;" Dr. D. J. Coleman, "The Alumni;" Dr. John E. Harris, "Graduating Class;" Hon. A. J. Montague, "The Doctor and the State;" Dr. Lewis S. McMurtry, Louisville, Ky., "Medical Education in the South;" Dr. William Warren Potter, Buffalo, N. Y., "The Surgeon in War."

At the conclusion of the responses to toasts Captain Frank Cunningham was introduced and sang the "Blue and the Gray" in a clear baritone voice, which proved a most appropriate conclusion to a delightful evening.

---

THE Buffalo Marine Hospital will soon be an accomplished fact if the signs at Washington are of any value in indicating results. The committee on inter-state and foreign commerce of which representative James S. Sherman is chairman, reported a bill to the House of Representatives on Monday, May 21, 1900, providing for the establishment of a Marine Hospital in Buffalo.

The bill was introduced early in the session by Hon. D. S. Alexander and provides for an expenditure for buildings and grounds of a sum not to exceed $125,oco. The necessity for such a hospital is beyond dispute, and it is to be hoped that no unnecessary delay will overtake the measure.

